# Dietary fiber intake, the gut microbiome, and chronic systemic inflammation in a cohort of adult men

**DOI:** 10.1186/s13073-021-00921-y

**Published:** 2021-06-17

**Authors:** Wenjie Ma, Long H. Nguyen, Mingyang Song, Dong D. Wang, Eric A. Franzosa, Yin Cao, Amit Joshi, David A. Drew, Raaj Mehta, Kerry L. Ivey, Lisa L. Strate, Edward L. Giovannucci, Jacques Izard, Wendy Garrett, Eric B. Rimm, Curtis Huttenhower, Andrew T. Chan

**Affiliations:** 1 Clinical and Translational Epidemiology Unit, Massachusetts General Hospital and Harvard Medical School, Boston, MA USA; 2 Division of Gastroenterology, Massachusetts General Hospital and Harvard Medical School, Boston, MA USA; 3 Department of Biostatistics, Harvard T.H. Chan School of Public Health, Boston, MA USA; 4 Department of Epidemiology, Harvard T.H. Chan School of Public Health, Boston, MA USA; 5 Department of Nutrition, Harvard T.H. Chan School of Public Health, Boston, MA USA; 6 Division of Public Health Sciences, Department of Surgery, Washington University School of Medicine, St Louis, MO USA; 7 Alvin J. Siteman Cancer Center, Washington University School of Medicine, St Louis, MO USA; 8 Division of Gastroenterology, Department of Medicine, Washington University School of Medicine, St Louis, MO USA; 9 Microbiome and Host Health Programme, South Australian Health and Medical Research Institute, North Terrace, Adelaide, SA 5000 Australia; 10 Department of Nutrition and Dietetics, College of Nursing and Health Sciences, Flinders University, Adelaide, Australia; 11 Division of Gastroenterology, University of Washington School of Medicine, Seattle, WA USA; 12 Channing Division of Network Medicine, Department of Medicine, Brigham and Women’s Hospital and Harvard Medical School, Boston, MA USA; 13 Department of Food Science and Technology, University of Nebraska-Lincoln, Lincoln, NE USA; 14 Fred and Pamela Buffett Cancer Center, University of Nebraska Medical Center, Omaha, NE USA; 15 School of Biological Sciences, University of Nebraska, Lincoln, NE USA; 16 Department of Immunology and Infectious Diseases, Harvard T.H. Chan School of Public Health, Boston, MA USA; 17 Department of Medicine, Harvard Medical School, Boston, MA USA; 18 Broad Institute of MIT and Harvard, Cambridge, MA USA

**Keywords:** Fiber, Chronic inflammation, Gut microbiome, *Prevotella copri*, Diet-microbiota interaction, Pectin, Metagenomics, Cohort

## Abstract

**Background:**

A higher intake of dietary fiber is associated with a decreased risk of chronic inflammatory diseases such as cardiovascular disease and inflammatory bowel disease. This may function in part due to abrogation of chronic systemic inflammation induced by factors such as dysbiotic gut communities. Data regarding the detailed influences of long-term and recent intake of differing dietary fiber sources on the human gut microbiome are lacking.

**Methods:**

In a cohort of 307 generally healthy men, we examined gut microbiomes, profiled by shotgun metagenomic and metatranscriptomic sequencing, and long-term and recent dietary fiber intake in relation to plasma levels of C-reactive protein (CRP), an established biomarker for chronic inflammation. Data were analyzed using multivariate linear mixed models.

**Results:**

We found that inflammation-associated gut microbial configurations corresponded with higher CRP levels. A greater intake of dietary fiber was associated with shifts in gut microbiome composition, particularly Clostridiales, and their potential for carbohydrate utilization via polysaccharide degradation. This was particularly true for fruit fiber sources (i.e., pectin). Most striking, fiber intake was associated with significantly greater CRP reduction in individuals without substantial *Prevotella copri* carriage in the gut, whereas those with *P. copri* carriage maintained stable CRP levels regardless of fiber intake.

**Conclusions:**

Our findings offer human evidence supporting a fiber-gut microbiota interaction, as well as a potential specific mechanism by which gut-mediated systemic inflammation may be mitigated.

**Supplementary Information:**

The online version contains supplementary material available at 10.1186/s13073-021-00921-y.

## Background

Chronic low-grade systemic inflammation has been associated with several diseases that collectively comprise the leading causes of death worldwide, including cardiovascular disease, cancer, diabetes mellitus, and chronic kidney disease [1]. Emerging evidence from animal models has demonstrated that diet plays a critical role in driving host inflammatory responses, predominantly through alterations in the composition and metabolic activities of the gut microbiome, which in turn influence downstream physiology such as intestinal permeability [2, 3]. However, with scant human population data, our understanding of the complex interplay between diet, specific microbes, and how they affect chronic systemic inflammation remains limited.

Numerous epidemiological studies have linked higher dietary fiber intake to reduced risk for several chronic inflammatory diseases [4], with the beneficial effects of fiber varying according to fiber types and their food sources [5–7]. For example, in a meta-analysis of several prospective cohort studies, insoluble fiber and fiber from cereals showed the strongest inverse association with risk of coronary heart disease [5]. Conversely for Crohn’s disease and diverticulitis, the greatest benefit came from fruit fiber [6, 7]. Furthermore, fibers are not mechanistically equivalent. Both in vitro and in vivo studies have shown that discrete structural differences of dietary fiber may induce distinct anti-inflammatory effects [8–10]. In one study, compared to inulin, apple-derived pectin had a highly specific influence on the gut microbiome composition and strongly promoted *Eubacterium eligens* [8]. However, despite these compelling findings, the mechanisms underlying these heterogeneous associations have not been fully explored.

Independent of health outcomes, decreased dietary fiber intake has also been associated with gut microbial responses. These include overall lower gut microbial diversity [11], specific taxonomic changes (particularly among microbes involved in fermentation and production of short-chain fatty acids (SCFAs)) [12], and the production of these metabolites themselves [13–15]. Meanwhile, recent experimental evidence has further demonstrated interactions between dietary fiber and gut microbiota composition in the pathogenesis of inflammation by impairing intestinal barrier function and increasing permeability [3, 16, 17]. For instance, mice fed a low-fiber diet had greater Proteobacteria carriage, increased permeability, and a reduced growth rate of the inner mucus layer, whereas *Bifidobacterium* or fiber could prevent these mucus aberrations [16]. In healthy individuals, dietary fiber interventions, particularly involving fructans and galacto-oligosaccharides that are highly fermentable, results in enriched abundance of *Bifidobacteria* and increased fecal concentrations of beneficial SCFAs, such as butyrate [9, 18].

We thus performed a population-based study to examine the relationship between dietary fiber intake, the gut microbiome, and chronic systemic inflammation. This employed a subcohort of 307 men, the Men’s Lifestyle Validation Study (MLVS), nested within the Health Professionals Follow-Up Study (HPFS). We assessed stool metagenomes and metatranscriptomes in association with long-term and recent dietary fiber intake from various food sources. This served to elucidate the mechanisms underlying the direct effects of fiber intake on the microbiome and their potential direct and indirect effects on circulating C-reactive protein (CRP), a validated and widely accepted marker of systemic inflammation. Mildly elevated CRP levels have been associated with risk of cardiovascular disease and other chronic inflammatory diseases among initially healthy population [19–21]. We found that dietary fiber from fruit sources in particular (e.g., pectin) was associated with gut microbiome composition and function, and CRP reduction with fiber intake was greater in individuals without *Prevotella copri* carriage.

## Methods

### Study population and stool sample collection

The Health Professionals Follow-Up Study (HPFS) is an ongoing prospective cohort study of 51,529 US male health professionals aged 40 to 75 years at enrollment in 1986 [22]. Participants have been followed biennially querying lifestyle, medical, and other health-related information, with a follow-up rate greater than 90% of available person-time. For this analysis, we used data from a sub-study of the HPFS, the Men’s Lifestyle Validation Study (MLVS), which consisted of 908 men aged 65 to 80 years who were free from coronary heart disease, stroke, cancer (except squamous or basal cell skin cancer), or major neurological diseases.

From 2012 to 2013, we recruited 307 men in the MLVS and collected longitudinal stool samples [23]. Participants were asked to provide stool samples from two consecutive bowel movements 24–72 h apart, followed approximately 6 months later by collection of a second, similar paired samples. Participants placed each bowel movement into a container with RNAlater and completed a questionnaire detailing the date and time of evacuation, Bristol stool scale, and other relevant information. Paired specimens were stored in the RNAlater fixative at ambient temperature, shipped overnight to the Broad Institute of MIT and Harvard, and stored immediately in −80°C freezers until nucleic acid extraction. The stool collection method has been detailed and validated previously and showed that self-collection stool using the fixatives provided statistically near-identical multi’omics data to fresh frozen samples according to the Human Microbiome Project-validated protocol [23–25].

As previously described [23], we used Illumina HiSeq sequencing paired-end (2 × 101 nucleotides) shotgun sequencing platform. DNA was extracted from 925 samples, in addition to RNA from a subset of 372 samples from 96 participants who provided stool samples during both sampling periods and did not report the use of antibiotics within the past year.

### Assessment of long-term and recent intake of dietary fiber

In the HPFS, dietary intake was assessed every 4 years since enrollment in 1986 with a validated, semi-quantitative Food Frequency Questionnaire (FFQ) [26]. Participants were asked how often they typically consumed each food of a standard portion size (e.g., 1 fresh apple or pear) during the past year, ranging from “almost never” to “≥ 6 times per day. Daily intake of each nutrient was calculated by multiplying the reported frequency of each food item by its nutrient content and summing across foods, followed by curator quality control. Fiber intake was calculated using the Associations of Official Analytical Chemists method (accepted by the US FDA and the World Health Organization for nutrition labeling purposes) [27]. We adjusted fiber intake for total caloric intake using the nutrient residual method [28]. FFQs have demonstrated good reproducibility and validity for assessing habitual intake; the correlation coefficient comparing dietary fiber assessment from FFQ with multiple 7-day dietary records is 0.68 [26]. To represent long-term intake, we calculated the cumulative average of dietary fiber intake based on seven FFQs prior to the stool collection from 1986 through 2010.

Participants in the MLVS were also administered 7-day dietary records recording recent diet contemporaneously with stool sample collections. Participants measured and reported gram weights for foods using a Primo Multifunction Kitchen Scale and ruler before and after eating, provided recipes of home-prepared foods, and returned labels of store-brand products. The Nutrition Data System for Research was used to derive over 150 nutrients and dietary constituents including dietary fiber intake [29].

### Taxonomic and functional profiling

Taxonomic and functional profiles were generated by using the bioBakery meta’omics workflow [30]. Sequencing reads were passed through the KneadData 0.3 quality control pipeline (http://huttenhower.sph.harvard.edu/kneaddata) to remove low-quality read bases and reads of human origin. Taxonomic profiling was performed using MetaPhIAn 2.6.0 (http://huttenhower.sph.harvard.edu/metaphlan2) [31], which classifies the metagenomic reads to taxonomies and yields relative abundances of taxa identified in the sample based on approximately 1 million clade-specific marker genes derived from 17,000 microbial genomes (corresponding to >13,500 bacterial and archaeal). We excluded microbial species that did not surpass minimum prevalence (10% of samples) and relative abundance (0.01%) threshold.

Metagenomes and metatranscriptomes were functionally profiled using HUMAnN 2.11.0 (http://huttenhower.sph.harvard.edu/humann) [32]. Briefly, for each sample, taxonomic profiling is used to identify detectable organisms. Reads are recruited to sample-specific pangenomes including all gene families in any detected microorganisms using Bowtie2 [33]. Unmapped reads are aligned against UniRef90 [34] using DIAMOND translated search [35]. Hits are counted per gene family and normalized for length and alignment quality. For calculating abundances from reads that map to more than one reference sequence, search hits are weighted by significance (alignment quality, gene length, and gene coverage). UniRef90 abundances from both the nucleotide and protein levels were then (1) mapped to level 4 enzyme commission (EC) nomenclature, (2) combined into structured pathways from MetaCyc [36], and (3) regrouped to carbohydrate-active enzymes (CAZy) [37]. More details about functional profiling in the MLVS have been described previously [23, 25].

Metatranscriptomic functional activity was assessed in 372 samples with both RNA and DNA data using RNA/DNA ratios. Owing to the compositionality of RNA and DNA measurements, the resulting ratio is relative to the mean transcript abundance of the entire microbial community. Thus, a ratio of 1 implies that the pathway is transcribed at the mean transcription abundance of all pathways in the microbial community. Infinite values of RNA/DNA ratios were imputed using 99% percentile of a given feature and values of 0 were imputed using half of the 1% percentile before log2 transformation. We excluded metagenomes and metatranscriptomes that did not surpass a minimum prevalence (10% for DNA and 20% for RNA/DNA ratio) and relative abundance (0.001% for DNA) threshold.

### Blood sample collection and measurement of CRP

The MLVS collected two fasting blood samples in blood tubes with liquid sodium heparin during the same period as fecal sample collection. After collection, tubes were placed in styrofoam containers with ice packs, returned to the laboratory via overnight courier, and centrifuged and aliquoted for storage in liquid nitrogen freezers (−130°C or colder). Plasma levels of high-sensitivity CRP were measured at the Franke lab at University of Hawaii using a Cobas MiraPlus clinical chemistry analyzer (Roche Diagnostics, Indianapolis, IN) and a latex particle enhanced immunoturbidimetry-based kit from Pointe Scientific (Lincoln Park, MI). We excluded samples with high-sensitivity CRP levels below (<0.01 mg/L) or above (>20 mg/dL), the detection limits.

### Data analysis

We paired longitudinal stool microbiome with concurrent blood measurements and dietary intake, resulting in 925 samples for taxonomic and metagenomic analyses and 372 samples for metatranscriptomic analyses. We used the Bray-Curtis dissimilarity metric to determine the differences of the taxonomic composition at the species level, functional potential (DNA), and functional activity (RNA/DNA ratio). We performed omnibus testing with permutational multivariate analysis of variance (PERMANOVA) of Bray-Curtis dissimilarities (999 permutations) to quantify the percentage of variance explained by age, lifestyle, diet, and clinical biomarkers. We identified microbial species and functions associated with dietary fiber intake and CRP using multivariate linear mixed model in MaAsLin 2 0.99.12 [38]. All models included each participant’s membership as a random effect to account for within-person variation and were also adjusted for covariates including age, recent antibiotic use, and total calorie intake:

$$ \mathrm{microbiome}\ \mathrm{features}\sim \mathrm{fiber}+\mathrm{age}+\mathrm{antibiotic}\ \mathrm{use}+\mathrm{calorie}\ \mathrm{intake}+\left(1\ |\ \mathrm{participant}\right) $$

We additionally adjusted for body mass index (BMI) in the model for CRP to delineate microbial species associated with chronic inflammation independent of adiposity. To examine an interaction between fiber intake and gut microbiome composition, we applied an equivalent multivariate linear mixed model that included a product term of fiber and presence of *P. copri* (fiber: *P. copri*) in addition to their main effects and evaluated the statistical significance of the product term:

$$ \mathrm{logCRP}\sim \mathrm{fiber}+P. copri+\mathrm{fiber}:P. copri+\mathrm{age}+\mathrm{antibiotic}\ \mathrm{use}+\mathrm{calorie}\ \mathrm{intake}+\left(1\ |\ \mathrm{participant}\right) $$

Overall type I error was controlled using the Benjamini-Hochberg false discovery rate (FDR). A corrected *p* value of <0.25 was considered as statistically significant, with some results limited to a subset for visualization and full results in the supplement.

## Results

### Study design and population

**Fig. 1:**
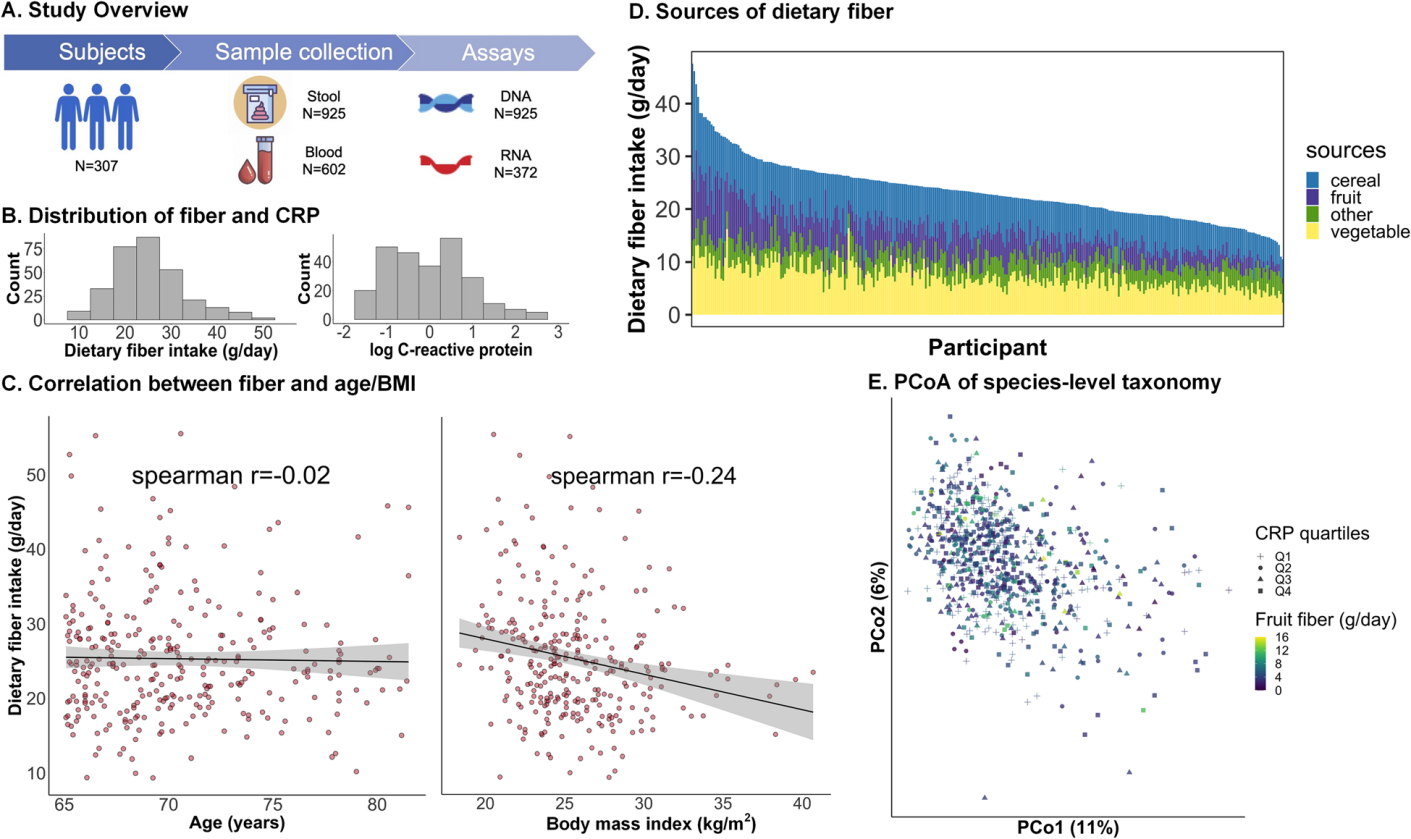
Linking the gut microbiome, dietary fiber, and systemic inflammation in a cohort of adult males. **A** 307 participants nested within the Health Professionals Follow-Up Study [23, 25] provided up to four stool samples with concurrent blood samples over a 6-month study period, generating 925 metagenomes from all participants and 372 metatranscriptomes from a subset of 96 selected because they provided stool at both sampling periods and did not report antibiotic use during the past year. **B** Overall recent dietary fiber intake and C-reactive protein (as a biomarker of systemic inflammation) levels were distributed representatively across this population. **C** Recent dietary fiber intake was inversely correlated with body mass index as expected (*r* =−0.24), but not correlated with age (*r* =−0.02). **D** Major food sources of fiber intake included cereals, vegetables, and fruits. **E** Principal coordinate analysis based on species-level Bray-Curtis dissimilarity decorated by quartiles of C-reactive protein and continuous fruit fiber intake suggested that fiber intake and CRP levels were not the overall largest sources of microbial community variability (other fiber subsets in Additional file 1: Figure S1)

We assessed diet-microbiome-inflammation interactions using existing data from the MLVS, a cohort of 307 generally healthy men nested in the HPFS (Fig. 1A), as described previously [23, 25]. The mean age of MLVS participants was 70.6±4.3 years. At the first collection, participants had a mean intake of dietary fiber of 25.3±8.1 g/d and a mean CRP level of 1.75 mg/dL (Fig. 1B). Recent intake of dietary fiber was inversely correlated with BMI as expected (*r* =−0.24), but not correlated with age (*r* =−0.02) (Fig. 1C). The source of dietary fiber was 33% from cereals, 21% from fruits, and 31% from vegetables (Fig. 1D). The majority of participants were physically active, did not smoke, and had normal stool consistency as represented by Bristol score; interestingly, these characteristics did not differ by overall fiber intake (Table 1).

**Table 1:** Characteristics of the 307 MLVS study participants according to quartiles of recent dietary fiber intake

	Quartiles of recent dietary fiber intake			
	1 (*n* = 77)	2 (*n* = 77)	3 (*n* = 77)	4 (*n* = 76)
Age, years	70.0 (4.1)	71.9 (4.5)	70.0 (4.0)	70.2 (4.1)
**Body mass index, kg/m**^**2**^	26.1 (3.2)	26.9 (4.5)	25.2 (2.9)	24.1 (3.3)
Physical activity, MET-hrs/week	48.2 (39.9)	39.3 (31.2)	52.4 (42.3)	51.1 (32.5)
Total energy intake, kcal/d	2363 (475)	2231 (481)	2312(453)	2337 (473)
**Total fiber intake, g/d**	16.6 (2.6)	22.0 (1.4)	26.4 (1.4)	36.2 (6.5)
**Cereal fiber, g/d**	6.2 (1.9)	6.9 (1.9)	7.7 (2.3)	9.5 (3.4)
**Fruit fiber, g/d**	3.1 (1.5)	4.4 (2.0)	5.4 (2.2)	6.7 (3.1)
**Vegetable fiber, g/d**	6.2 (2.4)	7.3 (2.2)	7.1 (2.2)	8.2 (2.1)
**Alcohol intake, %**				
Never	4.3	12.5	13.2	18.9
Rarely	17.0	14.3	15.1	17.0
1–6 times/week	38.3	37.5	43.4	34.0
Daily	36.2	32.1	26.4	30.2
More than daily	4.3	3.6	1.9	0
Antibiotic use, %	29.2	28.6	27.8	21.8
Probiotic use, %	4.3	3.6	11.5	5.8
Current smoker, %	2.6	0	1.3	0
Bristol score, %				
1–2, hard stool	14.6	18.2	7.4	12.7
3–5, normal stool	79.2	78.2	87.0	81.8
6–7, loose stool	6.3	3.6	5.6	5.5
Total cholesterol, mg/dL	186 (38)	182 (41)	186 (42)	178 (33)
HDL cholesterol, mg/dL	59.9 (13.7)	55.1 (15.0)	56.2 (15.3)	55.5 (12.9)
Total/HDL cholesterol ratio	3.2 (0.8)	3.4 (0.9)	3.4 (0.8)	3.3 (0.7)
C-reactive protein, mg/dL	2.1 (2.8)	1.9 (2.9)	1.3 (2.1)	1.7 (2.5)

Values are means (SD) for continuous variables and percentages for categorical variables. Variables that differed across quartiles of recent intake of dietary fiber are bolded (general linear model with F test for continuous variables and Mantel-Haenszel chi-squared test for categorical variables; *p* < 0.05)

### Baseline inter-individual variation in the gut microbiome dominates effects relative to dietary fiber intake

We included 925 metagenomes and 372 metatranscriptomes in our analyses [23]. A total of 139 microbial species were retained after quality control from MetaPhlAn 2 [31] and gene, transcript, and pathway functional profiles from DNA and RNA using HUMAnN 2 [32].

We first tested for associations between overall microbiome structure and our main variables of interest (fiber subsets and CRP) and covariates (Fig. 1E, Additional file 1: Figures S1 and S2). Using omnibus testing with PERMANOVA of Bray-Curtis dissimilarities, individual factors including age, lifestyle, diet, and clinical biomarkers only explained a minimal amount of the variation of the gut microbiome profile (all R^2^<0.01; Additional file 1: Figure S2). Among them, recent dietary fiber intake was the leading factor, explaining small but significant variance in taxonomic composition (R^2^=0.0095, *p* = 0.005) and functional potential (R^2^=0.0085, *p* = 0.001). Thus, neither fiber intake nor CRP levels alone were the main drivers of overall microbiome configurations, which were instead dominated by baseline inter-individual differences [25].

### Microbiome structure, primarily via *Prevotella copri* carriage, modifies the association between fiber intake and plasma CRP

**Fig. 2:**
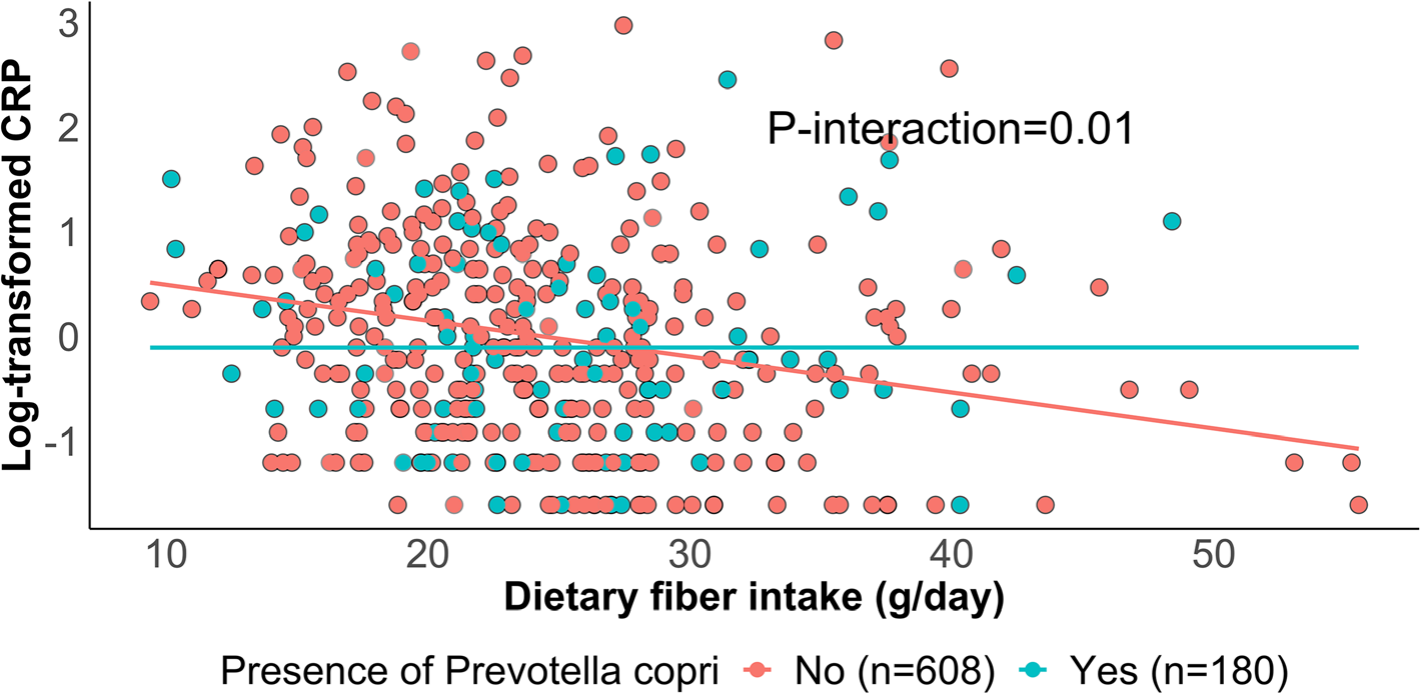
*Prevotella copri* carriage abrogates the protective effects of recent dietary fiber intake on C-reactive protein. Multivariate linear mixed models of log-transformed CRP were fit including recent fiber intake and *P. copri* carriage (binary), their interaction, accounting for participant membership as a random effect, and adjusting for age, recent antibiotic use, and total calorie intake. We excluded 137 samples with CRP values below or above the detection limits, and thus, 788 samples from 277 participants were included in this analysis. The relationship between dietary fiber and plasma CRP was significantly stronger among participants who did not carry *P. copri* (0 abundance)

We examined the possibility that the relationship between recent dietary fiber intake and CRP levels was not uniform across the population. Specifically, across the landscape of gut microbiome configurations (beta-diversity differences), we observed that individuals in the outgroup of high *Prevotella copri* carriers (a common subset of “typical” adult Westernized gut populations) did not appear to maintain the expected protective relationship between increased fiber and lower CRP. Consistent with other findings [39], 24.1% of samples in our study reliably carried *P. copri.* We tested this interaction quantitatively and found that the inverse association between dietary fiber and CRP was significantly stronger among participants who did not have *P. copri*, compared to those with *P. copri* carriage (P-interaction=0.01, using a model with a product term of the two in addition to covariates; Fig. 2). We tested the 10 most abundant species and found that *P. copri* was the only species whose abundance modified the association between fiber intake and CRP. We also found similar modulation effects of *P. copri* at each timepoint separately. For example, the Spearman correlation between recent fiber intake and CRP was **−**0.08 and **−**0.24 among samples with and without *P. copri* carriage at timepoint 1, and **−**0.04 and **−**0.31 at timepoint 2.

These results support that microbiome structure, primarily via *P. copri* carriage, likely modifies the effects of fiber intake in alleviating chronic inflammation. Relatedly, *P. copri* has previously shown both positive and negative influences on human health. Some studies have linked *P. copri* to improved glucose tolerance and insulin responses in fiber-rich diets [40, 41]. Others, in contrast, associated *P. copri* with insulin resistance and glucose intolerance as well as inflammatory diseases [42–44]. In combination with recent evidence that Westernization leads to reduced prevalence and genetic diversity of *P. copri* [39] and the much greater amount and diversity of plant-based dietary fiber sources in global diets, our results provide compelling novel evidence for chronic, systemic health consequences of gut microbial metabolism of dietary compounds.

### Individual members and functions in the microbiome are associated with recent and long-term fiber intake quantities and CRP

**Fig. 3:**
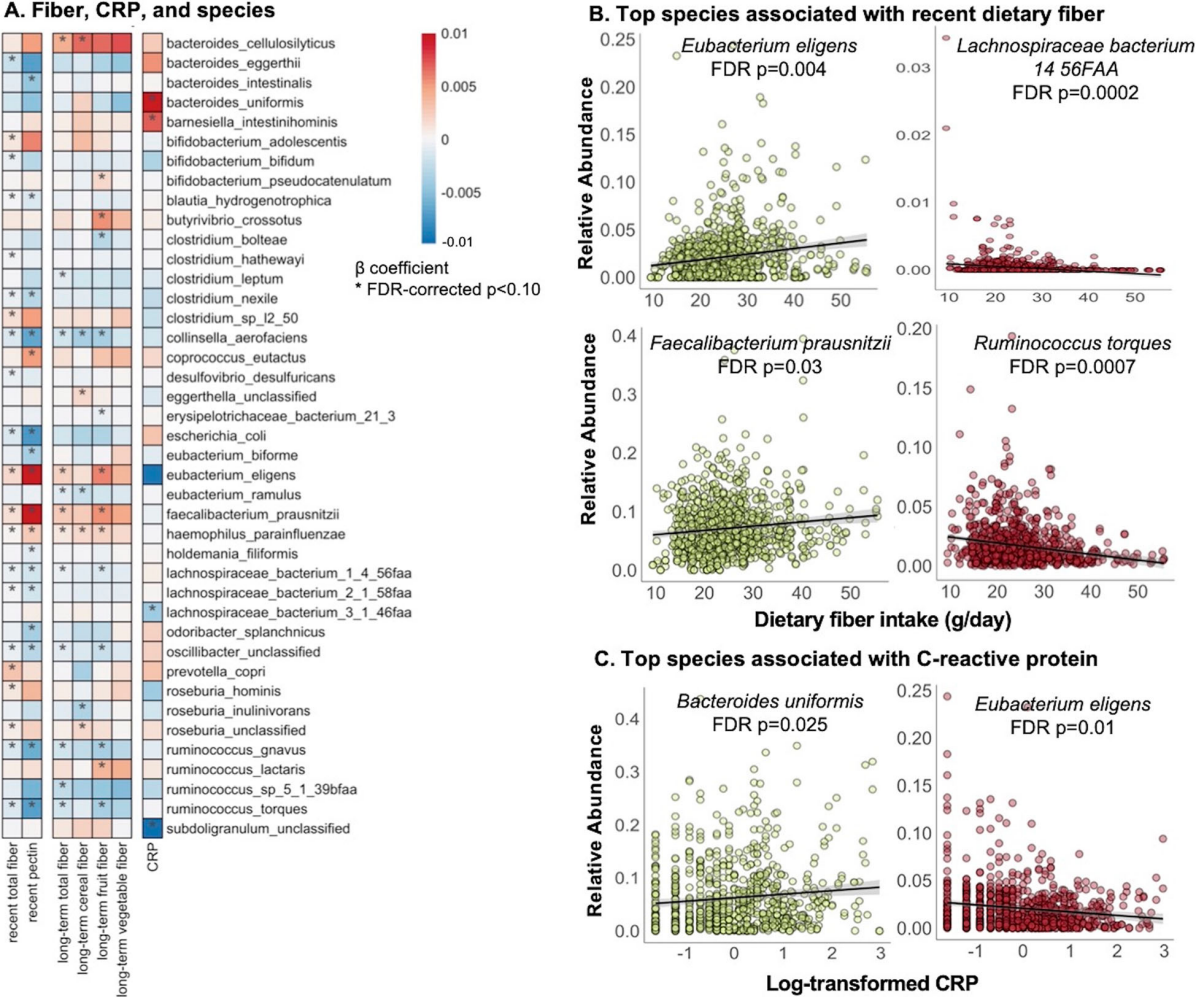
Species abundances significantly associated with C-reactive protein and dietary fiber intake. We included 925 metagenomic samples from 307 participants in this analysis. Comparisons used log-transformed CRP and fiber assessed as recent intake using both 7-day dietary records and long-term cumulative averages from Food Frequency Questionnaires over 1986-2010. **A** Significant associations between recent and long-term dietary fiber and CRP and metagenomic microbial species abundances using multivariate linear association testing (see the “Methods” section). Models were adjusted for age, recent antibiotics, and total calorie intake; models for CRP were additionally adjusted for body mass index. All species with FDR-corrected *p* < 0.25 are in Additional file 1: Figure S3 and Additional file 2: Table S2. **B** Raw (non-residualized) abundances for the species associated with recent dietary fiber intake and **C** CRP. Both recent and long-term higher dietary fibers were associated with shifts in individual microbial species such as Clostridiales. Greater microbial differences were observed in association with intake of pectin and fiber from fruits and, to a lesser extent, cereals, compared to vegetable fiber. Higher CRP levels corresponded with a generally inflammation-associated gut microbial configuration [45]

In the absence of overall microbiome shifts with fiber intake or CRP, we next identified individual microbial species associated with these variables using multivariate linear mixed model in MaAsLin 2 [38] (Fig. 3). All models included one fiber or inflammation outcome of interest, each participant’s membership as a random effect, and were also adjusted for covariates including age, recent antibiotic use, and total calorie intake. Consistent with previous studies [46], both recent and long-term higher dietary fiber were associated with shifts in Clostridiales, the major butyrate producers, including increases of *Eubacterium eligens*, *Faecalibacterium prausnitzii*, and genus *Roseburia*, but also decreases in *Clostridium*, *Lachnospiraceae*, and *Ruminococcus* spp. Increased fiber intake was also associated with increased relative abundances of *Haemophilus parainfluenzae* and *Bacteroides cellulosilyticus*. These associations remained robust despite additional adjustment for Bristol score and other lifestyle factors including alcohol intake and physical activity.

Greater microbial differences were observed in association with intake of pectin and fiber from fruits and, to a lesser extent, cereals, compared to vegetable fiber. For example, the findings mentioned above including positive associations with *E. eligens* and *F. prausnitzii* and inverse associations with *Lachnospiraceae* and *Ruminococcus* were largely driven by pectin and fruit fiber. The distinct chemical structures of dietary fibers lead to substantial variations in solubility and fermentability and subsequent effects on the microbial composition and functions [9, 10]. Pectin is a soluble dietary fiber rich in apples, pears, plums, and citrus fruits. It comprises a highly complex set of plant cell wall polysaccharides including homo-polygalacturonan, rhamnogalacturonan I, and rhamnogalacturonan II [47]. Our results were in line with reports from in vitro and animal studies that pectin induced influences on the gut microbiota composition, including increases of Clostridiales such as *F. prausnitzii* and a highly selective promotion of *E. eligens* as well as depletion of Bacteroidetes [48–50]. Associations of soluble and insoluble fiber with microbial species were similar (Additional file 1: Figure S3, Additional file 2: Table S2), possibly due to the challenge of differentiating the soluble vs. insoluble subtypes through available diet instruments [51].

In multivariate models for CRP, higher CRP was associated with enrichment of *B. uniformis*, *B. salyersale*, *Barnesiella intestinihominis*, and *Parabacteroides* independent of adiposity. In a previous analysis of 178 older adult subjects, CRP was positively associated with a metagenomically assembled *Bacteroides* co-abundance group [52]. However, unlike the positive association observed here with *P. distasonis* and *P. johnsonii*, the former was shown to alleviate obesity and metabolic dysfunctions via production of succinate and secondary bile acids in mice [53]. Higher CRP levels were also associated with depletion of *Lachnospiraceae bacterium 3 1 46FAA*, *E. eligens*, and *Bifidobacterium bifidum*, consistent with their anti-inflammatory effects as shown in experimental studies [50, 54, 55].

Dietary fiber intake in particular recent intake from pectin was also significantly associated with a large number of metagenomic functional pathways (Additional file 1: Figure S4, Additional file 2: Table S3) and features (Additional file 1: Figure S5, Additional file 2: Table S4) involved in the metabolism of carbohydrates and amino acids. A greater total fiber intake was associated with significant enrichment of PWY-7456 β-(1,4)-mannan degradation, P124 Bifidobacterium shunt, PWY-5104 L-isoleucine biosynthesis IV, and PWY-6305 putrescine biosynthesis, whereas the rest of pathways were generally depleted. A higher intake of total fiber and pectin was also associated with significantly enriched expression of EC 3.2.1.4, an endoglucanase (hydrolyzing of (1,4)-beta-d-glucan linkages in cellulose) and EC 2.4.1.1 (glycogen phosphorylase), both of which play a role in catalyzing the degradation of glycans or polysaccharides.

### Potential biochemical contributors to microbe-specific selection pressures from dietary fiber

To specifically investigate fiber intake in relation to functional capacity and activity relating to carbohydrate utilization, we further mapped gene families into carbohydrate-active enzymes (CAZy) [37]. CAZy covers enzymes catalyzing the breakdown, biosynthesis, or modification of carbohydrates and glycoconjugates including glycoside hydrolases (GHs), glucosylTransferases (GTs), polysaccharide lyases (PLs), carbohydrate esterases (CEs), auxiliary activities (AAs), and non-catalytic carbohydrate-binding modules (CBMs). We excluded enzymes that did not surpass a minimum prevalence (10% of samples for CAZy DNA and 20% for RNA/DNA ratio) and relative abundance (0.001% for CAZy DNA) threshold, resulting in 134 DNA CAZys and 121 RNA/DNA features in the analysis

**Fig. 4:**
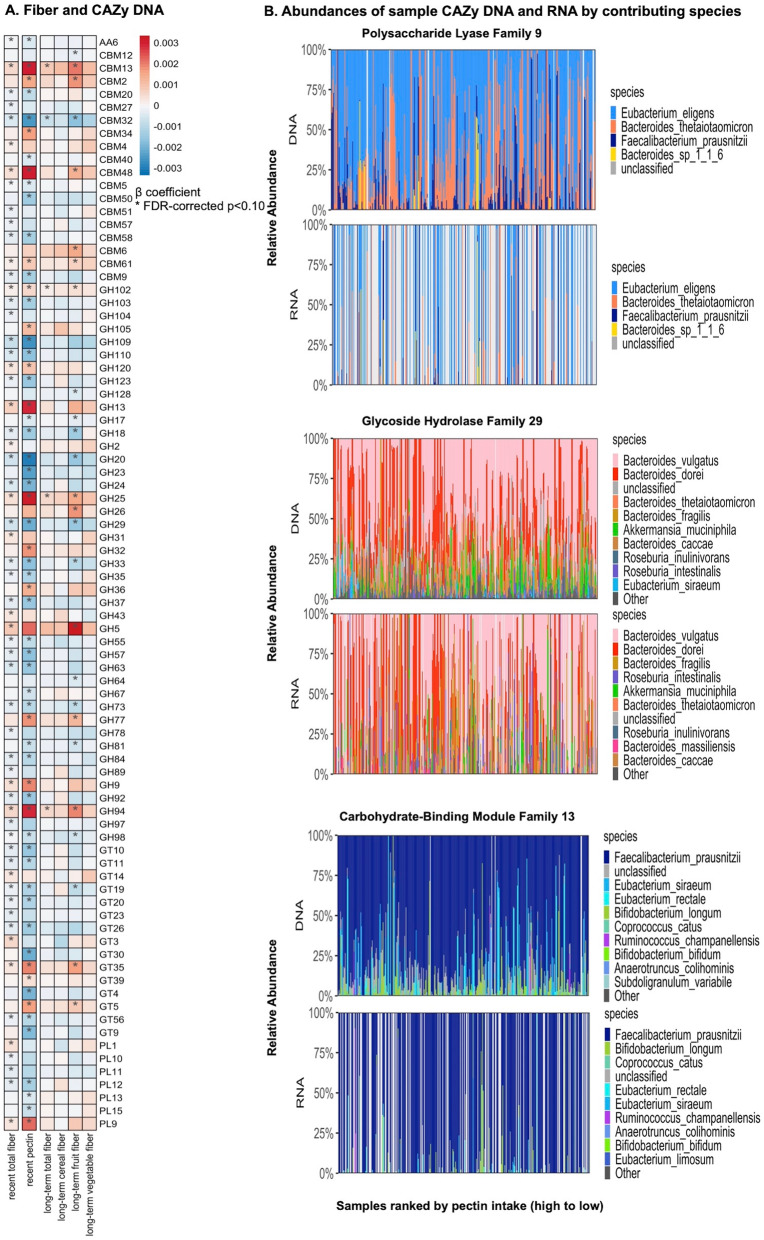
CAZy and dietary fiber intake. **A** Significant associations between recent and long-term dietary fiber and CAZy DNA abundances using multivariate linear association testing (see the “Methods” section, Additional file 2: Table S5). Comparisons used fiber assessed as recent intake using both 7-day dietary records and long-term cumulative averages from Food Frequency Questionnaires over 1986–2010. Models were adjusted for age, recent antibiotics, and total calorie intake. **B** Abundances of metagenomes and metatranscriptomes of polysaccharide lyase family 9 (PL9), glycoside hydrolase family 29 (GH29), and carbohydrate-binding module family 13 (CBM13) by contributing species and samples, with species ranked by mean relative abundance, and samples ranked by pectin intake. We included 925 metagenomes and 341 metatranscriptomes in this analysis. A total of 84 CAZys metagenomically associated with dietary fiber in particular pectin and fruit fiber. Positive associations were observed particularly for several CAZy DNA and RNA/DNA ratios contributed by Clostridiales species

We identified a total of 84 CAZys metagenomically associated with dietary fiber—again, particularly pectin and fruit fiber—using multivariate linear testing (Fig. 4A, Additional file 2: Table S5). Concordant with the chemical structure of pectin consisting of repeated units of α -(1-4)-linked d-galacturonic acid, and the fermentation requirement of pectinase, we detected an enrichment of PL9 strongly positively correlated with pectin intake. PL9 covers enzymes including pectate lyase (EC 4.2.2.2), exopolygalacturonate lyase (EC 4.2.2.9), thiopeptidoglycan lyase (EC 4.2.2.-), and rhamnogalacturonan endolyase (EC 4.2.2.23) and participates in the degradation of homogalacturonan. In our samples, expression of PL9 was primarily contributed by *E. eligens*, followed by *B. thetaiotaomicron*, *F. prausnitzii*, and *B. sp. 1_1_6* (Fig. 4B). An increase of GH25, carried by diverse species of *Eubacterium*, *Bacteroides*, and *Faecalibacterium*, was also strongly associated with fiber and pectin intake. Meanwhile, fiber and pectin also showed inverse associations with some features, such as GH29, contributed largely by *Bacteroides* species and involved in degradation of other glycan targets. Finally, some other enzyme families that were contributed mostly by Clostridiales, such as CBM13, were also associated with a greater intake of fiber and pectin.

We additionally evaluated the associations of recent dietary fiber and pectin with copy-number normalized transcript levels of CAZys, i.e., RNA/DNA ratios (Additional file 1: Figure S6, Additional file 2: Table S6, see the “Methods” section). Interestingly, this recapitulated the positive association between PL9 and dietary fiber. There was also a trend towards positive correlation between pectin intake and PL9, although not achieving statistical significance. These results provide additional support for the gene family’s functional role in polysaccharide degradation in vivo.

## Discussion

Here, we have demonstrated one of the first explicit interaction relationships by which a specific component of the gut microbiome (*P. copri*) modifies a dietary exposure (fiber intake) with a well-established marker of systemic inflammation (plasma C-reactive protein levels). In addition to this interaction effect, direct effects of dietary fiber intake on the gut microbiome generally increased Clostridiales, which also play a pivotal role in regulating localized and systemic inflammation [56]. These microbial alterations also varied among specific fiber sources, with the greatest effects deriving from pectin and fruit fiber. For instance, abundances of *E. eligens* and *F. prausnitzii* as well as their functions in the degradation of polysaccharides were enriched in participants with greater dietary fiber and, especially, pectin intake. We also linked individual microbial signatures to chronic systemic inflammation. These findings collectively offer novel human evidence supporting a variety of fiber-gut-microbiome interactions relevant to chronic systemic inflammation.

Specifically, in our population, *P. copri* carriage eliminated the strongly protective effects of increased fiber intake on systemic inflammation, with *P. copri* carriers distributed across a range of generally modest CRP levels and non-carriers varying between higher and lower extremes according to fiber intake. The impact of *P. copri* on human health overall is still controversial, as conflicting results have been reported among different populations and phenotypes. As a fiber-degrader, *Prevotella* was positively associated with production of SCFAs (e.g., propionate) [57, 58] and could, for example, provide host benefit by improved glucose metabolism in response to a high-fiber diet [40, 41]. Conversely, *P. copri* has been associated with chronic inflammatory conditions such as rheumatoid arthritis [42, 43] and insulin resistance and glucose intolerance [44]. Strain-level heterogeneity and distinct clades of the *P. copri* complex may contribute to its functional diversity and some of these apparent phenotypic contradictions [59, 60]. For instance, genetically diverse *P. copri* isolates utilize distinct sets of polysaccharides from dietary plant sources [59]. The combination of *P. copri* diversity, fiber type and amount diversity, and the gradual, multi-generational loss of *P. copri* clades from Westernized populations could account for the complexity of this interaction [39]. Our findings suggest that *P. copri* could in principle have both direct effects on systemic inflammation, as well as opposing, indirect effects caused by reduced bioavailability of fermentable fibers or other fermentation products to other microbes. Additional investigation is thus needed to functionally characterize the influence of *P. copri* on modulating dietary effects on inflammation, health, and host-microbe coevolution.

An additional intriguing result from this study was the specificity of many fiber-microbiome influences to fruit fibers and pectin. As a major soluble fiber component in the plant cell wall, particularly in fruits and vegetables, pectin serves as the nutritional niche for some groups of bacteria, such as *B. thetaiotaomicron* [61], *F. prausnitzii* [49], and *E. eligens* [8]. It is likely that the chemical complexity of pectin relative to other fiber sources facilitates its capacity to nourish diverse microbial communities [62]. Polysaccharide utilization loci that orchestrate the detection, sequestration, enzymatic digestion, and transport of complex carbohydrates have been identified in most gut-resident species, especially among the Bacteroidetes [63]. However, knowledge of the impact of pectin in particular on the gut microbial communities is still limited and has been restricted to in vitro and animal studies. To our knowledge, we for the first time identified pectin-induced alterations in gut microbiota composition and functional capabilities and subsequent impact in inflammation in a human population study. These results suggest that pectin intake may exert a selection pressure on the gut microbiota leading to the predominance of organisms that degrade pectic polysaccharides and an enhancement of functional activities specifically based on their utilization. This supports the notion that gut microbial strains are highly specialized, particularly with respect to carbon source utilization and products, and can evolve and adapt over the course of an adult lifetime to utilize a unique subset of complex polysaccharides in a personalized, individual-specific manner [61].

At least one additional recent study, using a distinct population and methodology, found potentially similar between-subject variation in fiber sources with respect to the microbiome. There, significant agreement between microbiome composition and fiber-source diversity was observed for fruits and grains, but not for vegetables or legumes [64]. Such heterogeneity according to fiber sources might be explained not only by the distinct chemical structures of fibers in each type of food [10], but also by other fruit-specific bioactive compounds such as polyphenols (flavonoids, phenolic acids, and carotenoids [65]) and even cooking (raw vs. cooked plant foods) [66]. Our population-based investigation does not distinguish between these potential mechanisms, for which in vitro studies and randomized controlled trials of specific fibers are better suited (although these cannot, conversely, assess the long-term effects of dietary fiber). Likewise, as an observational study, we cannot be definitive about causality and although the fiber-microbiome-inflammation association was robust despite adjustment for many variables, we cannot rule out the potential for residual confounding. Finally, since our study only included older adult men in the US, we are cautious about generalizability to other populations, especially, younger and non-Western populations in whom relevant dietary or microbial components may be quite distinct). Thus, we plan to validate these findings in additional cohorts with information on diet, the gut microbiome, and health outcomes.

## Conclusions

As one of the only sustainable long-term influences on the gut microbiome and chronic health, dietary interactions and interventions are a key strategy to mitigate chronic inflammation. Our findings will benefit from further investigation of the specific mechanisms by which *P. copri* mediates dietary biochemistry and host inflammation, as well as the specific routes by which pectin directly influences other gut microbiome members. An understanding of these distinct effects of dietary fibers, pectin, and how they are transformed and utilized by microbial communities would pave the way forward for development of personalized fiber-based interventions for the prevention of chronic inflammatory diseases.

## Supplementary Information

**Additional file 1: Figures S1**-**S6.**

**Additional file 2: Tables S1**-**S6.**

## Data Availability

All the metadata from the Health Professionals Follow-Up Study are available through a request for external collaboration and upon approvals of a letter of intent and a research proposal. Details for how to request an external collaboration with the Health Professionals Follow-Up Study can be found at https://sites.sph.harvard.edu/hpfs/for-collaborators/. Source code that generates the figures and tables is available at https://github.com/biobakery/Fiber-Microbiome-Inflammation [67].

## Abbreviations

CRP: C-reactive protein
SCFA: Short-chain fatty acids
MLVS: Men’s Lifestyle Validation Study
HPFS: Health Professionals Follow-Up Study
FFQ: Food Frequency Questionnaire
EC: Enzyme commission
CAZy: Carbohydrate-active enzymes
FDR: False discovery rate
