# Supplementary material for: Dietary fiber intake, the gut microbiome, and chronic systemic inflammation in a cohort of adult men
Source: Genome Med. 2021 Jun 17;13:102. doi: 10.1186/s13073-021-00921-y (PMC8212460; doi:10.1186/s13073-021-00921-y)
Supplement: Supplementary file 1 — Additional file 1: Figures S1-S6. [file 13073_2021_921_MOESM1_ESM.pdf]

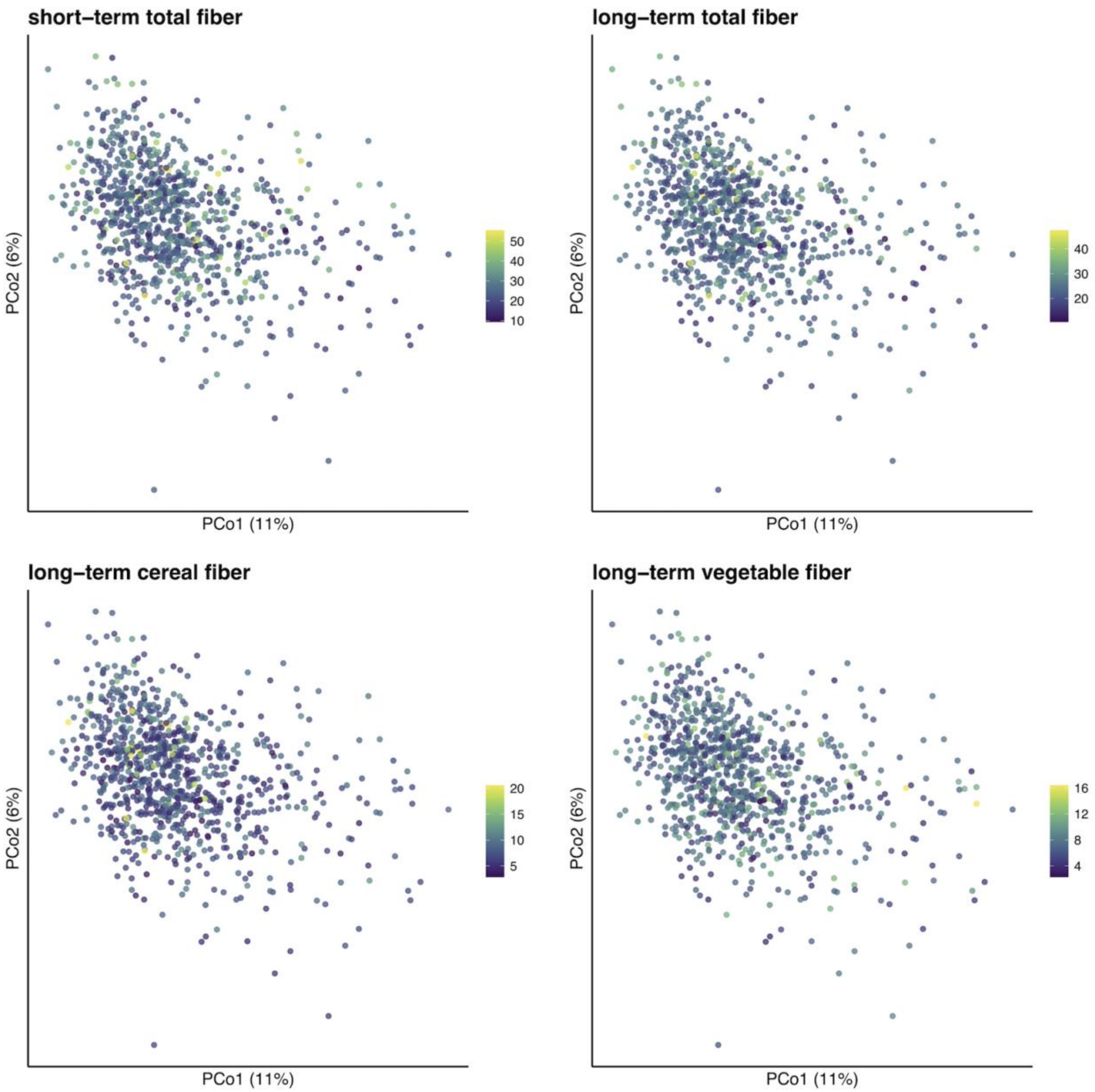
short−term total fiber
long−term total fiber
long−term cereal fiber
long−term vegetable fiber
PCo1 (11%)
PCo2 (6%)
50
40
30
20
10
40
30
20
20
15
10
5
16
12
8
4

Figure S1: **Principal coordinate analysis based on species-level Bray-Curtis dissimilarity.** Long-term fiber intake was represented by a cumulative average of dietary intake values based on seven validated, semi-quantitative food frequency questionnaires from 1986 through 2010. In 2012-2013, 307 participants provided up to four stool samples over a six-month study period with concurrent blood samples and assessments of recent dietary intake using 7-day dietary records. Shotgun metagenomic and metatranscriptomic sequencing and profiling were conducted, with bacteria abundance determined using MetaPhIAn 2. A total of 139 microbial species were included in the analysis after filtering by minimum prevalence (>10%) and relative abundance (>0.01%). Dietary fiber did not explain overall microbial communities.

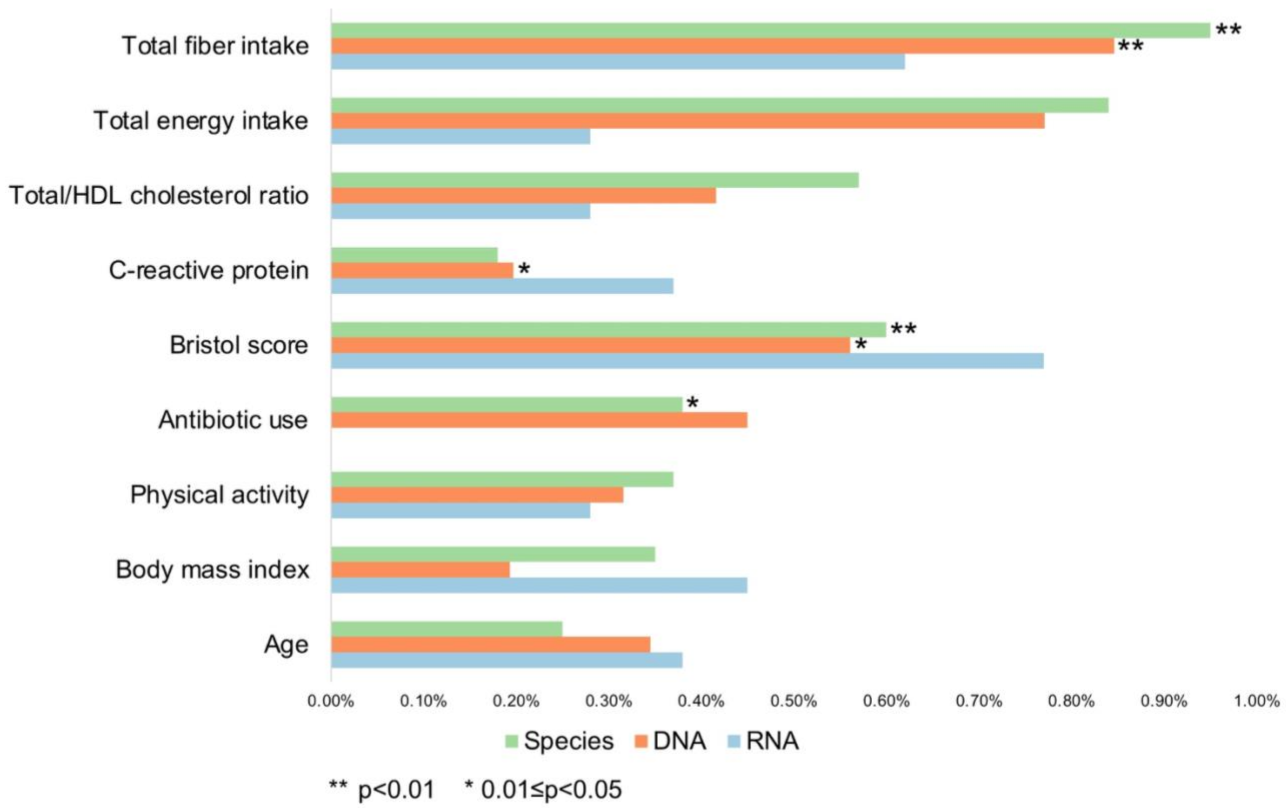

Figure S2: **Omnibus testing with permutational analysis of variance testing (PERMANOVA) of Bray-Curtis dissimilarities (999 permutations).** In our study, 307 generally healthy men (mean age: 70.6±4.3 years) provided up to four stool samples over a six-month study period with concurrent blood samples. They also provided assessments of recent dietary intake using 7-day dietary records and lifestyle information via questionnaires. A total of 925 metagenomes and 372 metatranscriptomes were included in the analysis. Bacteria abundance was determined using MetaPhlAn 2, with 139 microbial species retained after filtering by minimum prevalence (>10%) and relative abundance (>0.01%). Functional profiles of DNA and RNA reads were generated using HUMAnN 2. Individual factors including age, lifestyle, diet and clinical biomarkers only explained a minimal amount of the variation of the gut microbiome profile (all R^2<0.01; **Additional file 2: Table S1**). Among them, fiber intake was the leading factor explaining small but significant variance in taxonomic composition (R^2=0.0095, p=0.005) and functional potential (R^2=0.0085, p=0.001).

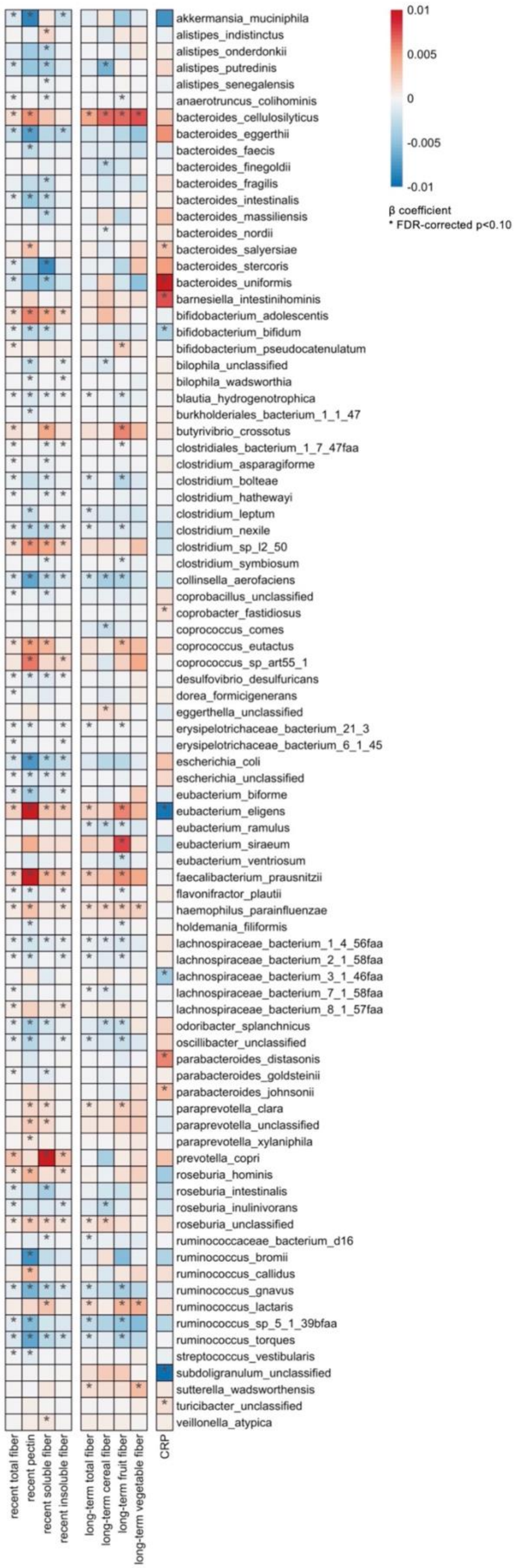
akkermansia_muciniphila
alistipes_indistinctus
alistipes_onderdonkii
alistipes_putredinis
alistipes_senegalensis
anaerotruncus_colihominis
bacteroides_cellulosilyticus
bacteroides_eggerthii
bacteroides_faecis
bacteroides_finegoldii
bacteroides_fragilis
bacteroides_intestinalis
bacteroides_massiliensis
bacteroides_nordii
bacteroides_salyersiae
bacteroides_stercoris
bacteroides_uniformis
barnesiella_intestinihominis
bifidobacterium_adolescentis
bifidobacterium_bifidum
bifidobacterium_pseudocatenulatum
bilophila_unclassified
bilophila_wadsworthia
blautia_hydrogenotrophica
burkholderiales_bacterium_1_1_47
butyrivibrio_crossotus
clostridiales_bacterium_1_7_47faa
clostridium_asparagiforme
clostridium_bolteae
clostridium_hathewayi
clostridium_leptum
clostridium_nexile
clostridium_sp_l2_50
clostridium_symbiosum
collinsella_aerofaciens
coprobacillus_unclassified
coprobacter_fastidiosus
coprococcus_comes
coprococcus_eutactus
coprococcus_sp_art55_1
desulfovibrio_desulfuricans
dorea_formicigenerans
eggerthella_unclassified
erysipelotrichaceae_bacterium_21_3
erysipelotrichaceae_bacterium_6_1_45
escherichia_coli
escherichia_unclassified
eubacterium_biforme
eubacterium_eligens
eubacterium_ramulus
eubacterium_siraeum
eubacterium_ventriosum
faecalibacterium_prausnitzii
flavonifractor_plautii
haemophilus_parainfluenzae
holdemania_filiformis
lachnospiraceae_bacterium_1_4_56faa
lachnospiraceae_bacterium_2_1_58faa
lachnospiraceae_bacterium_3_1_46faa
lachnospiraceae_bacterium_7_1_58faa
lachnospiraceae_bacterium_8_1_57faa
odoribacter_splanchnicus
oscillibacter_unclassified
parabacteroides_distasonis
parabacteroides_goldsteinii
parabacteroides_johnsonii
paraprevotella_clara
paraprevotella_unclassified
paraprevotella_xylaniphila
prevotella_copri
roseburia_hominis
roseburia_intestinalis
roseburia_inulinivorans
roseburia_unclassified
ruminococcaceae_bacterium_d16
ruminococcus_bromii
ruminococcus_callidus
ruminococcus_gnavus
ruminococcus_lactaris
ruminococcus_sp_5_1_39bfaa
ruminococcus_torques
streptococcus_vestibularis
subdoligranulum_unclassified
sutterella_wadsworthensis
turicibacter_unclassified
veillonella_atypica
recent total fiber
recent pectin
recent soluble fiber
recent insoluble fiber
long-term total fiber
long-term cereal fiber
long-term fruit fiber
long-term vegetable fiber
CRP
0.01
0.005
0
-0.005
-0.01
β coefficient
* FDR-corrected p<0.10

Figure S3: Species abundances significantly associated with C-reactive protein and dietary fiber intake (FDR-corrected p<0.25). We included 925 metagenomic samples from 307 participants in this analysis. Comparisons used log-transformed CRP and fiber assessed as recent intake using both 7-day dietary records and long-term cumulative averages from food frequency questionnaires over 1986-2010. Significant associations between recent and long-term dietary fiber and CRP and metagenomic microbial species abundances were obtained using multivariate linear association testing (**Methods**). Models were adjusted for age, recent antibiotics, and total calorie intake; models for CRP were additionally adjusted for body mass index. Both recent and long-term higher dietary fiber were associated with shifts in individual microbial species such as Clostridiales. Greater microbial differences were observed in association with intake of pectin and fiber from fruits and to a lesser extent, cereals, compared to vegetable fiber. Higher CRP levels were associated with an inflammation-associated gut microbial configuration. Data are also shown in **Additional file 2: Table S2**.

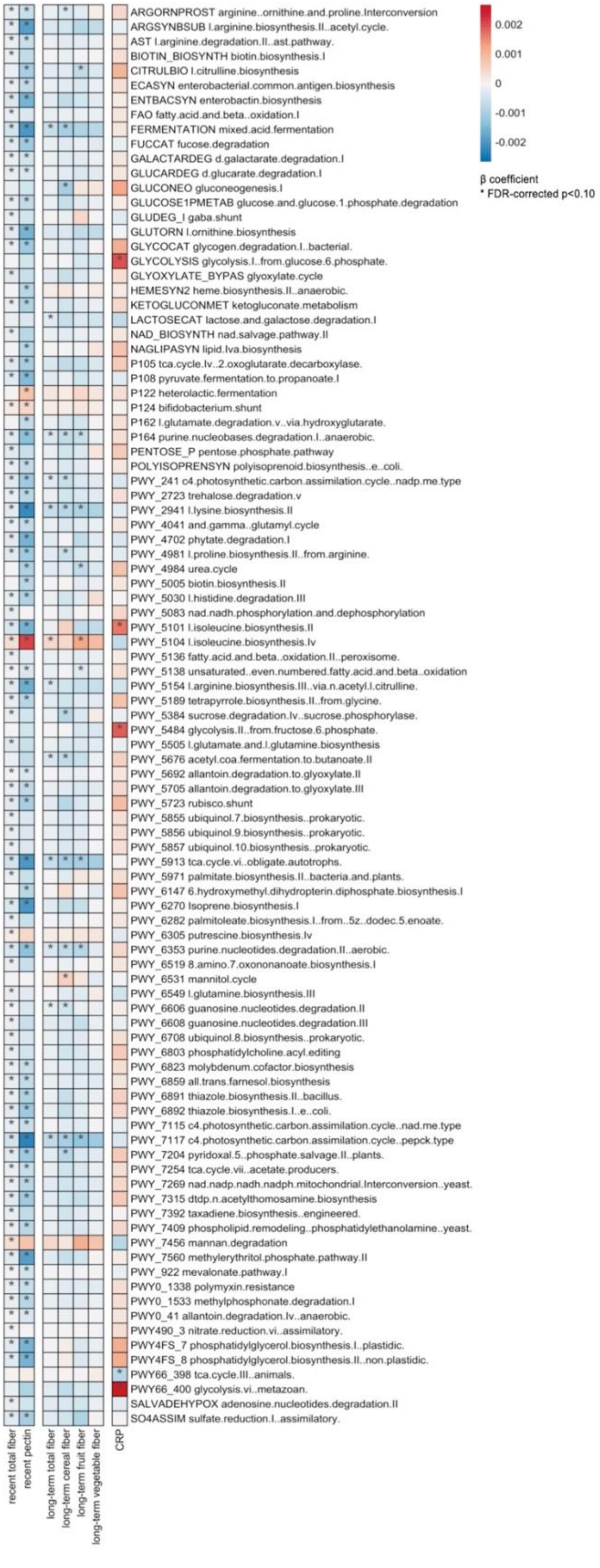
ARGORNPROST arginine..ornithine.and.proline.Interconversion
ARGSYNBSUB l.arginine.biosynthesis.II..acetyl.cycle.
AST l.arginine.degradation.II..ast.pathway.
BIOTIN_BIOSYNTH biotin.biosynthesis.I
CITRULBIO l.citrulline.biosynthesis
ECASYN enterobacterial.common.antigen.biosynthesis
ENTBACSYN enterobactin.biosynthesis
FAO fatty.acid.and.beta..oxidation.I
FERMENTATION mixed.acid.fermentation
FUCCAT fucose.degradation
GALACTARDEG d.galactarate.degradation.I
GLUCARDEG d.glucarate.degradation.I
GLUCONEO gluconeogenesis.I
GLUCOSE1PMETAB glucose.and.glucose.1.phosphate.degradation
GLUDEG_I gaba.shunt
GLUTORN l.ornithine.biosynthesis
GLYCOCAT glycogen.degradation.I..bacterial.
GLYCOLYSIS glycolysis.I..from.glucose.6.phosphate.
GLYOXYLATE_BYPAS glyoxylate.cycle
HEMESYN2 heme.biosynthesis.II..anaerobic.
KETOGLUCONMET ketogluconate.metabolism
LACTOSECAT lactose.and.galactose.degradation.I
NAD_BIOSYNTH nad.salvage.pathway.II
NAGLIPASYN lipid.Iva.biosynthesis
P105 tca.cycle.Iv..2.oxoglutarate.decarboxylase.
P108 pyruvate.fermentation.to.propanoate.I
P122 heterolactic.fermentation
P124 bifidobacterium.shunt
P162 l.glutamate.degradation.v..via.hydroxyglutarate.
P164 purine.nucleobases.degradation.I..anaerobic.
PENTOSE_P pentose.phosphate.pathway
POLYISOPRENSYN polyisoprenoid.biosynthesis..e..coli.
PWY_241 c4.photosynthetic.carbon.assimilation.cycle..nadp.me.type
PWY_2723 trehalose.degradation.v
PWY_2941 l.lysine.biosynthesis.II
PWY_4041 and.gamma..glutamyl.cycle
PWY_4702 phytate.degradation.I
PWY_4981 l.proline.biosynthesis.II..from.arginine.
PWY_4984 urea.cycle
PWY_5005 biotin.biosynthesis.II
PWY_5030 l.histidine.degradation.III
PWY_5083 nad.nadh.phosphorylation.and.dephosphorylation
PWY_5101 l.isoleucine.biosynthesis.II
PWY_5104 l.isoleucine.biosynthesis.Iv
PWY_5136 fatty.acid.and.beta..oxidation.II..peroxisome.
PWY_5138 unsaturated..even.numbered.fatty.acid.and.beta..oxidation
PWY_5154 l.arginine.biosynthesis.III..via.n.acetyl.l.citrulline.
PWY_5189 tetrapyrrole.biosynthesis.II..from.glycine.
PWY_5384 sucrose.degradation.Iv..sucrose.phosphorylase.
PWY_5484 glycolysis.II..from.fructose.6.phosphate.
PWY_5505 l.glutamate.and.l.glutamine.biosynthesis
PWY_5676 acetyl.coa.fermentation.to.butanoate.II
PWY_5692 allantoin.degradation.to.glyoxylate.II
PWY_5705 allantoin.degradation.to.glyoxylate.III
PWY_5723 rubisco.shunt
PWY_5855 ubiquinol.7.biosynthesis..prokaryotic.
PWY_5856 ubiquinol.9.biosynthesis..prokaryotic.
PWY_5857 ubiquinol.10.biosynthesis..prokaryotic.
PWY_5913 tca.cycle.vi..obligate.autotrophs.
PWY_5971 palmitate.biosynthesis.II..bacteria.and.plants.
PWY_6147 6.hydroxymethyl.dihydropterin.diphosphate.biosynthesis.I
PWY_6270 Isoprene.biosynthesis.I
PWY_6282 palmitoleate.biosynthesis.I..from..5z..dodec.5.enoate.
PWY_6305 putrescine.biosynthesis.Iv
PWY_6353 purine.nucleotides.degradation.II..aerobic.
PWY_6519 8.amino.7.oxononanoate.biosynthesis.I
PWY_6531 mannitol.cycle
PWY_6549 l.glutamine.biosynthesis.III
PWY_6606 guanosine.nucleotides.degradation.II
PWY_6608 guanosine.nucleotides.degradation.III
PWY_6708 ubiquinol.8.biosynthesis..prokaryotic.
PWY_6803 phosphatidylcholine.acyl.editing
PWY_6823 molybdenum.cofactor.biosynthesis
PWY_6859 all.trans.farnesol.biosynthesis
PWY_6891 thiazole.biosynthesis.II..bacillus.
PWY_6892 thiazole.biosynthesis.I..e..coli.
PWY_7115 c4.photosynthetic.carbon.assimilation.cycle..nad.me.type
PWY_7117 c4.photosynthetic.carbon.assimilation.cycle..pepck.type
PWY_7204 pyridoxal.5..phosphate.salvage.II..plants.
PWY_7254 tca.cycle.vii..acetate.producers.
PWY_7269 nad.nadp.nadh.nadph.mitochondrial.Interconversion..yeast.
PWY_7315 dtdp.n.acetylthomosamine.biosynthesis
PWY_7392 taxadiene.biosynthesis..engineered.
PWY_7409 phospholipid.remodeling..phosphatidylethanolamine..yeast.
PWY_7456 mannan.degradation
PWY_7560 methylerythritol.phosphate.pathway.II
PWY_922 mevalonate.pathway.I
PWY0_1338 polymyxin.resistance
PWY0_1533 methylphosphonate.degradation.I
PWY0_41 allantoin.degradation.Iv..anaerobic.
PWY490_3 nitrate.reduction.vi..assimilatory.
PWY4FS_7 phosphatidylglycerol.biosynthesis.I..plastidic.
PWY4FS_8 phosphatidylglycerol.biosynthesis.II..non.plastidic.
PWY66_398 tca.cycle.III..animals.
PWY66_400 glycolysis.vi..metazoan.
SALVADEHYPOX adenosine.nucleotides.degradation.II
SO4ASSIM sulfate.reduction.I..assimilatory.
recent total fiber
recent pectin
long-term total fiber
long-term cereal fiber
long-term fruit fiber
long-term vegetable fiber
CRP
0.002
0.001
0
-0.001
-0.002
β coefficient
* FDR-corrected p<0.10

Figure S4. Metagenomic pathways significantly associated with C-reactive protein and dietary fiber intake. We included 925 metagenomic samples from 307 participants in this analysis. Comparisons used log-transformed CRP and fiber assessed as recent intake using both 7-day dietary records and long-term cumulative averages from food frequency questionnaires over 1986-2010. Significant associations between recent and long-term dietary fiber and CRP and metagenomic pathways were obtained using multivariate linear association testing (**Methods**). Models were adjusted for age, recent antibiotics, and total calorie intake; models for CRP were additionally adjusted for body mass index. Dietary fiber intake in particular recent intake from pectin was significantly associated with a large number of metagenomic functional pathways involved in the metabolism of carbohydrates and amino acids. Data are also shown in **Additional file 2: Table S3**.

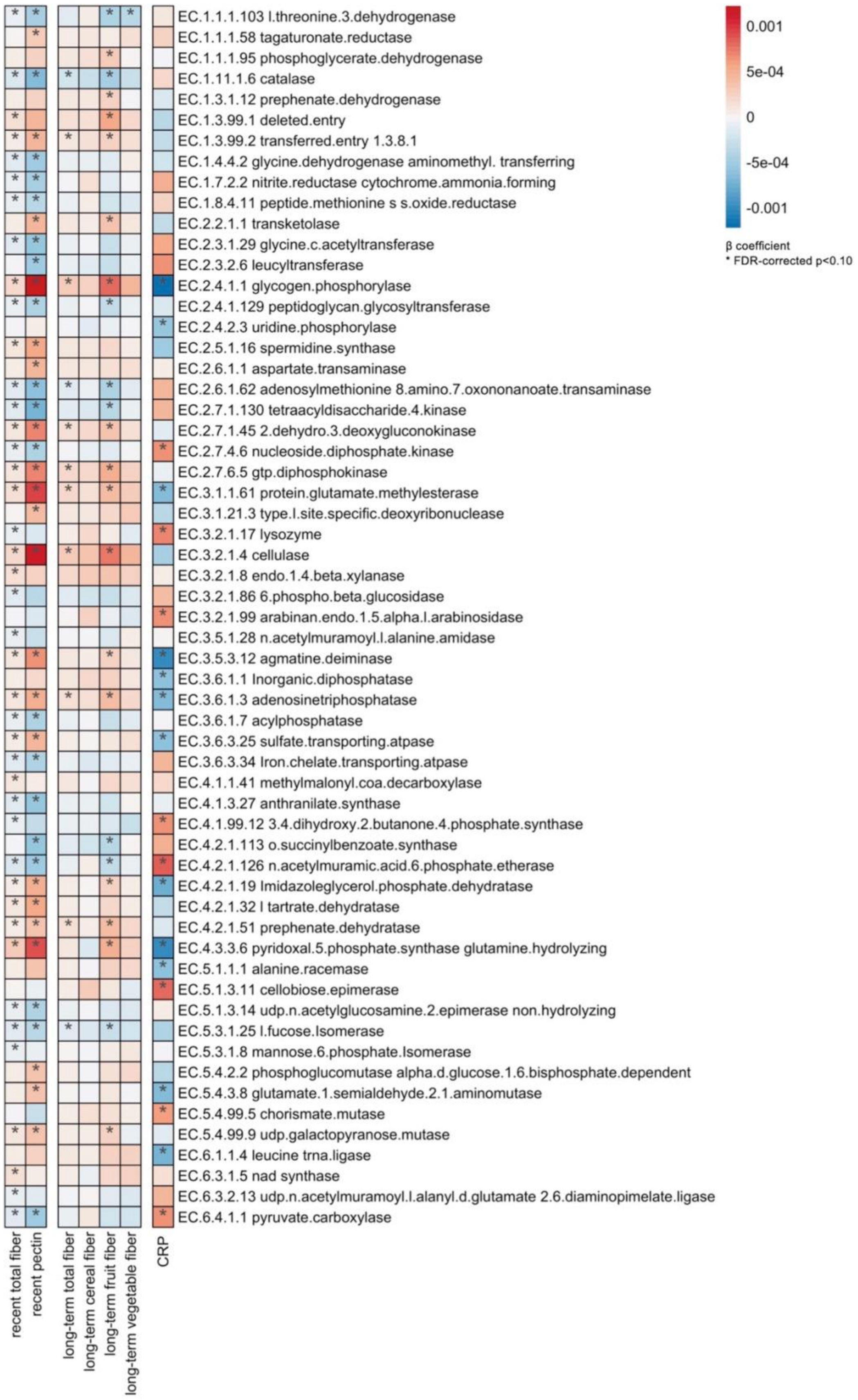
EC.1.1.1.103 l.threonine.3.dehydrogenase
EC.1.1.1.58 tagaturonate.reductase
EC.1.1.1.95 phosphoglycerate.dehydrogenase
EC.1.11.1.6 catalase
EC.1.3.1.12 prephenate.dehydrogenase
EC.1.3.99.1 deleted.entry
EC.1.3.99.2 transferred.entry 1.3.8.1
EC.1.4.4.2 glycine.dehydrogenase aminomethyl. transferring
EC.1.7.2.2 nitrite.reductase cytochrome.ammonia.forming
EC.1.8.4.11 peptide.methionine s s.oxide.reductase
EC.2.2.1.1 transketolase
EC.2.3.1.29 glycine.c.acetyltransferase
EC.2.3.2.6 leucyltransferase
EC.2.4.1.1 glycogen.phosphorylase
EC.2.4.1.129 peptidoglycan.glycosyltransferase
EC.2.4.2.3 uridine.phosphorylase
EC.2.5.1.16 spermidine.synthase
EC.2.6.1.1 aspartate.transaminase
EC.2.6.1.62 adenosylmethionine 8.amino.7.oxononanoate.transaminase
EC.2.7.1.130 tetraacyldisaccharide.4.kinase
EC.2.7.1.45 2.dehydro.3.deoxygluconokinase
EC.2.7.4.6 nucleoside.diphosphate.kinase
EC.2.7.6.5 gtp.diphosphokinase
EC.3.1.1.61 protein.glutamate.methylesterase
EC.3.1.21.3 type.I.site.specific.deoxyribonuclease
EC.3.2.1.17 lysozyme
EC.3.2.1.4 cellulase
EC.3.2.1.8 endo.1.4.beta.xylanase
EC.3.2.1.86 6.phospho.beta.glucosidase
EC.3.2.1.99 arabinan.endo.1.5.alpha.l.arabinosidase
EC.3.5.1.28 n.acetylmuramoyl.l.alanine.amidase
EC.3.5.3.12 agmatine.deiminase
EC.3.6.1.1 Inorganic.diphosphatase
EC.3.6.1.3 adenosinetriphosphatase
EC.3.6.1.7 acylphosphatase
EC.3.6.3.25 sulfate.transporting.atpase
EC.3.6.3.34 Iron.chelate.transporting.atpase
EC.4.1.1.41 methylmalonyl.coa.decarboxylase
EC.4.1.3.27 anthranilate.synthase
EC.4.1.99.12 3.4.dihydroxy.2.butanone.4.phosphate.synthase
EC.4.2.1.113 o.succinylbenzoate.synthase
EC.4.2.1.126 n.acetylmuramic.acid.6.phosphate.etherase
EC.4.2.1.19 Imidazoleglycerol.phosphate.dehydratase
EC.4.2.1.32 l tartrate.dehydratase
EC.4.2.1.51 prephenate.dehydratase
EC.4.3.3.6 pyridoxal.5.phosphate.synthase glutamine.hydrolyzing
EC.5.1.1.1 alanine.racemase
EC.5.1.3.11 cellobiose.epimerase
EC.5.1.3.14 udp.n.acetylglucosamine.2.epimerase non.hydrolyzing
EC.5.3.1.25 l.fucose.Isomerase
EC.5.3.1.8 mannose.6.phosphate.Isomerase
EC.5.4.2.2 phosphoglucomutase alpha.d.glucose.1.6.bisphosphate.dependent
EC.5.4.3.8 glutamate.1.semialdehyde.2.1.aminomutase
EC.5.4.99.5 chorismate.mutase
EC.5.4.99.9 udp.galactopyranose.mutase
EC.6.1.1.4 leucine trna.ligase
EC.6.3.1.5 nad synthase
EC.6.3.2.13 udp.n.acetylmuramoyl.l.alanyl.d.glutamate 2.6.diaminopimelate.ligase
EC.6.4.1.1 pyruvate.carboxylase
recent total fiber
recent pectin
long-term total fiber
long-term cereal fiber
long-term fruit fiber
long-term vegetable fiber
CRP
0.001
5e-04
0
-5e-04
-0.001
β coefficient
* FDR-corrected p<0.10

Figure S5. Microbial functional potential abundances significantly associated with C-reactive protein and dietary fiber intake. We included 925 metagenomic samples from 307 participants in this analysis. Comparisons used log-transformed CRP and fiber assessed as recent intake using both 7-day dietary records and long-term cumulative averages from food frequency questionnaires over 1986-2010. Significant associations between recent and long-term dietary fiber and CRP and abundances of 100 most abundant, uncorrelated metagenomic enzyme commissions were obtained using multivariate linear association testing (**Methods**). Models were adjusted for age, recent antibiotics, and total calorie intake; models for CRP were additionally adjusted for body mass index. Both recent and long-term dietary fiber were significantly associated with a large number of metagenomic functional features involved in metabolism of carbohydrates, amino acids, vitamins, and other compounds. Greater differences were observed associated with pectin and fruit fiber. Data are also shown in **Additional file 2: Table S4**.

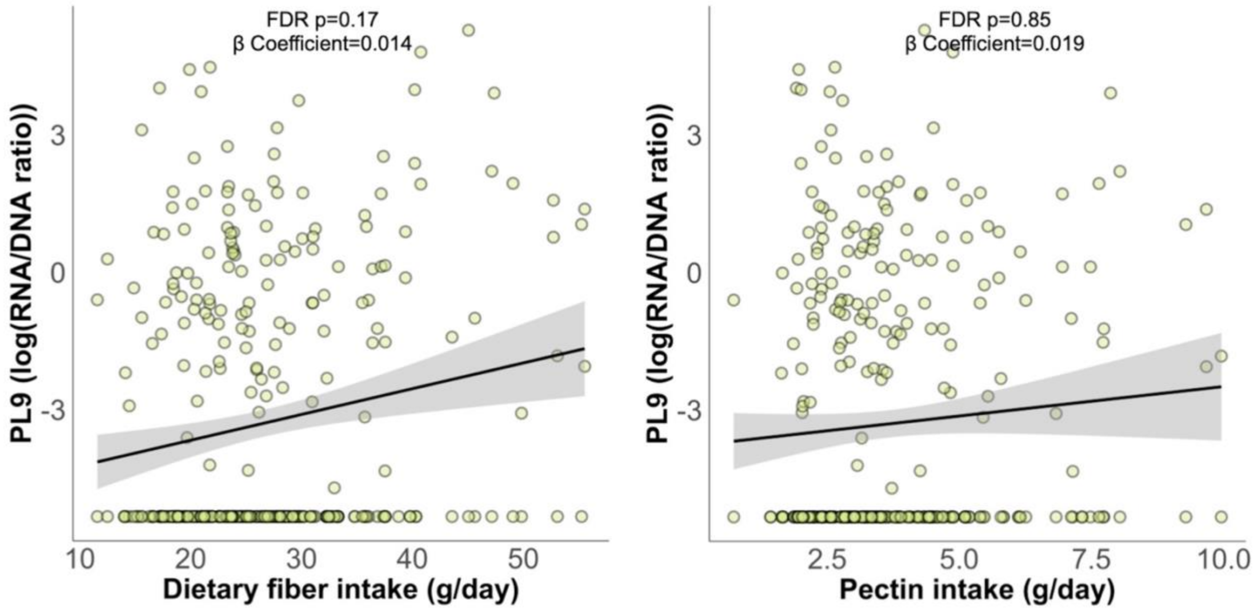

Figure S6: Functional activity of CAZy polysaccharide lyase family 9 (PL9) associated with recent dietary fiber/pectin intake. We included 372 samples with both metatranscriptomes and metagenomes from a subset of 96 participants selected because they provided stool at both sampling periods and did not report antibiotic use during the past year. Multivariate linear mixed models were adjusted for age, recent antibiotics, and total calorie intake. Functional activity of PL9 was represented by RNA/DNA ratio and log2 transformed. Positive associations were observed between functional activity of PL9 and dietary intake of fiber or pectin. All results for multivariable-adjusted associations between fiber and CAZy RNA/DNA ratios are shown in **Additional file 2: Table S6**.
